# Fluorescein-based monitoring of RNA N^6^-methyladenosine at single-nucleotide resolution

**DOI:** 10.1093/jmcb/mjaa057

**Published:** 2020-10-16

**Authors:** Xiao-Min Liu, Shen Wang, Xianqing Gan, Shu-Bing Qian, Jun Zhou

**Affiliations:** 1 School of Life Science and Technology, China Pharmaceutical University, Nanjing 210009, China; 2 State Key Laboratory of Natural Medicines, China Pharmaceutical University, Nanjing 210009, China; 3 Division of Nutritional Sciences, Cornell University, Ithaca, NY 14853, USA

**Dear Editor,**

N^6^-methyladenosine (m^6^A) emerges as an abundant chemical modification on RNAs, which enriches in distinct internal regions linked to divergent aspects of RNA fate (Zhao et al., 2017). Transcriptome-wide mapping of m^6^A sites using high-throughput sequencing enables comparative analysis of cellular m^6^A dynamics on particular RNAs under both physiological and stress conditions (Dominissini et al., 2012; Meyer et al., 2015), yet is confined to low resolution of coverage and indistinction of adjacent m^6^A sites. Broad research has focused on developing sensitive and reliable approaches to probe m^6^A status on individual transcript (Li et al., 2016). A DNA polymerase identified from *Thermus thermophilus* (*Tth* pol) is in favor of incorporating thymidine opposite unmodified A over m^6^A (Harcourt et al., 2013). The specific feature of this enzyme allows determining the locations of m^6^A in cellular rRNA and mRNA transcribed from exogenous plasmid. SCARLET enables accurate identification and quantification of m^6^A in endogenous mRNA and long noncoding RNA (lncRNA) (Liu et al., 2013). Nonetheless, these methods require radioisotope labeling that may limit their widespread applicabilities. Recently, two groups screened novel m^6^A-selective DNA ligases, which were able to accurately determine the m^6^A locus in mRNA and lncRNA from total RNA or polyA-RNA (Liu et al., 2018; Xiao et al., 2018). Both approaches avoid radioactive labeling but potentially generate unspecific signals induced by other modifications. Here, we present a refined approach termed site-specific monitoring of m^6^A via reverse transcription (SMART) using fluorescein-labelled dUTP (fluorescein-dUTP), which allows identification of innate m^6^A status at specific site of both *in vitro* synthesized and endogenous RNAs without transcript amplification. SMART combines m^6^A antibody crosslinking with fluorescein-dUTP incorporation during primer extension. In detail, synthesized or isolated RNA is incubated with m^6^A antibody followed by crosslinking with UV light at 254 nm wavelength. After crosslinking, the antibody‒m^6^A conjugation could efficiently impede the incorporation of fluorescein-dUTP during reverse transcription (RT). The fluorescent RT products are visualized by the imager after separation on the TBE-urea gel. The low fluorescence intensity of transcribed products at expected size would reflect high methylated fraction of specific adenosine on RNAs (Figure 1A).

**Figure 1:**
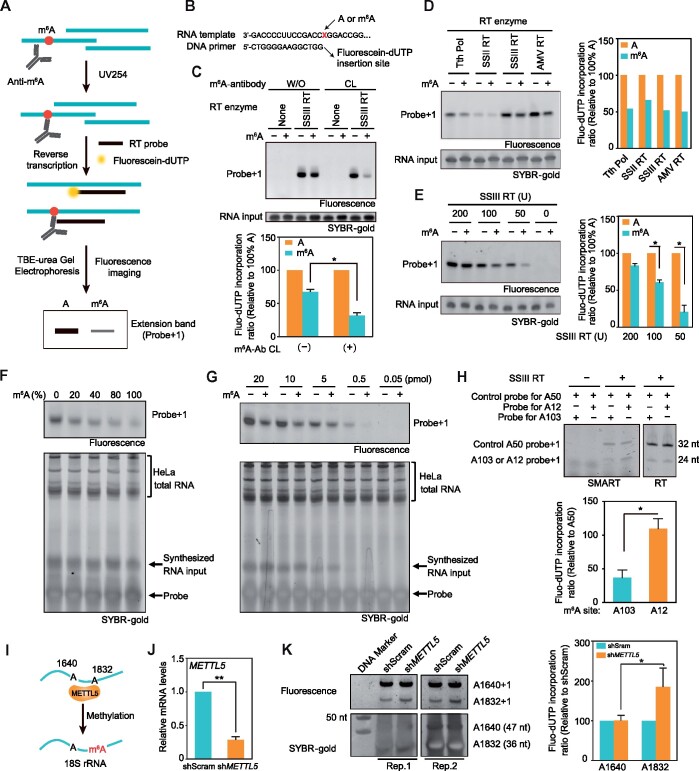
SMART-based detection of m^6^A at single adenosine site. (**A**) Schematic diagram of SMART. RNAs are incubated with anti-m^6^A antibody followed by crosslinking with 254 nm UV light. Antibody–RNA complexes are subjected to RT assay. The extension bands (probe + 1) are separated on a TBE-urea gel, and the signal is visualized by fluorescence gel imager. The covalently bound m^6^A antibody on the RNA impedes the incorporation of fluorescein-dUTP. (**B**) Sequences of RNA template and DNA RT probe used in **C**–**G**. (**C**) Comparison of SuperScript III-mediated RT with (CL) and without (W/O) m^6^A antibody incubation coupled with crosslinking. (**D**) Indicated reverse transcriptases were used in SMART assay with synthesized RNA. (**E**) Indicated doses of SuperScript III were used in SMART assay with synthesized RNA. The ‘probe + 1’ band signals were normalized to the signal from SYBR-gold-stained synthesized RNA input in the quantification panel in **C**–**E**. (**F**) Synthesized RNAs with varied ratios of m^6^A:A (250 ng) and 1 µg cellular RNA from HeLa cells were mixed and used in SMART assay. (**G**) Indicated amounts of synthesized RNA containing or not containing m^6^A were incubated with 1 µg total RNA from HeLa cells for SMART assay. (**H**) SMART (left) and regular RT (right) assays to probe m^6^A at three candidate sites of *Hspa1a* mRNA 5′UTR. The ‘probe + 1’ band signals at A103 or A12 were normalized to the signal at known non-methylated control site A50 in the quantification panel. (**I**) Schematic diagram showing METTL5-mediated methylation on A1832 of 18S ribosomal RNA. A1640 serves as a non-methylated control site. (**J** and **K**) HeLa cells with knockdown of *METTL5* were generated for SMART-based detection of m^6^A at two sites A1640 and A1832 of 18S rRNA. The ‘probe + 1’ band signal at A1640 or A1832 in sh*METTL5* cells was normalized to the signal in shScram cells in the quantification panel. Data in bar graphs are mean ± SD (*n *=* *3, except *n *=* *2 in **C**), unpaired Student’s *t*-test, *P <* *0.05, **P <* *0.01.

Unlike other epitranscriptomic codes, m^6^A marks located on RNA are not able to efficiently block the transcription elongation or generate readable mutations during RT. However, crosslinking of m^6^A-containing RNA with specific antibodies would produce a certain amount of truncated cDNAs during RT (Linder et al., 2015). With the availability of this principle, we initially assessed the feasibility of SuperScrip III-mediated SMART using a pair of synthetic RNA oligos harboring GGAC or GGm^6^AC (Figure 1B). As shown in Figure 1C and Supplementary Figure S1, the RT signal marked as ‘probe + 1’ produced by GGm^6^AC RNA with crosslinking was significantly weaker (28% relative to GGAC) than that without crosslinking (69% relative to GGAC), suggesting that antibodies fixed to m^6^A residue post crosslinking could substantially block the incorporation of fluorescein-dUTP. To broaden the availability of SMART, we examined inhibitory effects of m^6^A on RT efficiency using other widely used reverse transcriptases, including *Tth* pol, SuperScripII, and AMV. All the transcriptases exhibited retarded catalytic activities to various degrees when replacing A with m^6^A in the RNA template (Figure 1D). To further optimize the reaction conditions, different doses of SuperScrip III were used for SMART assay. While 200 U SuperScrip III catalyzed 80% fluorescein-dUTP incorporation at methylated adenosine, the ‘probe + 1’ RT product was reduced to only 20% in the presence of 50 U SuperScrip III, both compared to non-modified templates (100%) (Figure 1E;Supplementary Figure S2). Collectively, these results suggest that SMART assay is able to probe m^6^A on synthesized RNAs site-specifically and not confined to the specific nature of enzymes involved in the RT reaction.

To reasonably estimate the performance of SMART approach, we next examined whether the optimized version was suitable for quantitative detection of m^6^A deposition at specific loci. Different ratios of GGAC with GGm^6^AC RNA templates were mixed (0%, 20%, 40%, 80%, and 100%) and used for SMART reactions. The ‘probe + 1’ product abundance of SMART was in linear proportion to the m^6^A ratio in the RNA samples (Supplementary Figure S3A). To mimic the cellular condition, the assay was performed in a mixture of synthesized RNA with 1 µg HeLa cell total RNA. Notably, the RT signal was decreased in linear proportion along with increased methylated ratio of synthesized RNA (Figure 1F;Supplementary Figure S3B). Furthermore, SMART still enabled reliable detection of relative m^6^A levels when as little as ∼0.5 pmol target RNA was present in 1 µg cellular RNA (Figure 1G). This result implies that SMART assay bears commendable sensitivity to modified fraction of individual adenosine. Likewise, this encouraging result prompted us to examine whether SMART is applicable for monitoring cellular m^6^A at desired sites of RNA. We previously reported that 5′UTR methylation of *Hspa1a* transcripts facilitated cap-independent translation initiation (Zhou et al., 2015). During heat shock response, m^6^A at A103 site of *Hspa1a* mRNA underwent dynamic changes. To further confirm the existence of m^6^A at the specific locus, we designed a group of primers to probe methylation status at two individual adenosines of *Hspa1a* transcript, A103 and A12, both of which are located within the consensus DRACH motif. A50 served as a control site due to its location beyond the consensus sequence. As *Hspa1a* mRNA expresses at negligible levels under normal condition, MEF cells were exposed to heat stress at 42°C for 1 h and recovered at 37°C for 2 h. Transcript abundance of *Hspa1a* was rapidly increased after recovery. We conducted SMART assay for candidate site A103 or A12 together with A50 in the same reaction, thus the RT signal produced at A103 or A12 site could be normalized to that of A50 site (0% m^6^A). A50 exhibited comparable ‘probe + 1’ RT signal in two separate reactions, while A103 generated much weaker RT product signal than A12 (Figure 1H, left panel). This variant pattern was not observed in regular RT reactions without antibody crosslinking (Figure 1H, right panel), indicating the presence of m^6^A on *Hspa1a* A103 after heat shock response. To further verify the feasibility of SMART, we tended to choose more candidate sites, among which A1832 in 18S ribosomal RNA is well-identified (Liu et al., 2013). Recent studies demonstrated that METTL5 is the core methyltransferase catalyzing the m^6^A of A1832 (Figure 1I; Van Tran et al., 2019; Ignatova et al., 2020). We therefore generated HeLa cells with knockdown of *METTL5* (Figure 1J). The ‘probe + 1’ signal of A1832 was dramatically increased in response to METTL5 depletion (Figure 1K), while the signal from control site A1640 bore negligible changes. Collectively, these results provide evidence that SMART is capable of guiding site-specific measurement of m^6^A in mammalian cells. As the length of probes potentially affects the RT efficiency (Figure 1K), SMART may not be an ideal approach for comparative measurement of m^6^A levels on different sites within the same transcript.

In summary, we present a user-friendly approach to identify or validate m^6^A status of both *in vitro* synthesized and endogenous RNAs at single-nucleotide resolution. SMART couples m^6^A antibody crosslinking with fluorescein-dUTP incorporation during primer elongation. By visualizing the fluorescent signal of transcribed products at expected size, we found that the antibody‒m^6^A conjugation post crosslinking on the RNA could efficiently impede the incorporation of dUTP in m^6^A dose-dependent manner. Although the antibody is able to recognize m^6^Am as well, the location-specific probe could help distinguish SMART signals generated by internal m^6^A and terminal m^6^Am, which are located in distinct regions of the transcript (Linder et al., 2015). Using SMART approach, we validated the enrichment of m^6^A at A103 on *Hspa1a* 5′UTR after heat shock stress and further confirmed the methylation on A1832 of 18S ribosomal RNA installed by METTL5. This reliable SMART is radioisotope-free and easily applicable in regular labs. The precise comparative analysis of m^6^A under different conditions would help us better understand molecular mechanisms underlying m^6^A biogenesis as well as m^6^A-modulated cellular events.

*[Supplementary material is available at Journal of Molecular Cell Biology online. This work was supported by grants from the National Natural Science Foundation of China (81974527), the Natural Science Foundation of Jiangsu Province, China (BK20190533), and State Key Laboratory of Natural Medicines, China Pharmaceutical University (SKLNMZZRC201809) to J.Z., from US National Institutes of Health (R01GM1222814 and R21CA227917) and HHMI Faculty Scholar (55108556) to S.-B.Q., and from China Pharmaceutical University Research Start-up Fund (3150040072) to X.-M.L. J.Z., X.-M.L., and S.-B.Q. conceived the project. X.-M.L. and J.Z. performed most of the experiments and wrote the manuscript. S.W. and X.G. helped with cell culture and data collection. All authors discussed the results and edited the manuscript.]*

## Supplementary Material

mjaa057_Supplementary_Data
