# Supplementary material for: Fluorescein-based monitoring of RNA N6-methyladenosine at single-nucleotide resolution
Source: J Mol Cell Biol. 2020 Oct 16;13(4):325–8. doi: 10.1093/jmcb/mjaa057 (PMC8339360; doi:10.1093/jmcb/mjaa057)
Supplement: mjaa057_Supplementary_Data [file mjaa057_supplementary_data.pdf]

Supplementary Information

Fluorescein-based monitoring of RNA N^6-methyladenosine at single-nucleotide resolution

Xiao-Min Liu[1*], Shen Wang[1], Xianqing Gan[1], Shu-Bing Qian[3] and Jun Zhou[1, 2*]

[1] School of Life Science and Technology, China Pharmaceutical University, Nanjing, Jiangsu 210009, China.

[2] State Key Laboratory of Natural Medicines, China Pharmaceutical University, Nanjing, Jiangsu 210009, China.

[3] Division of Nutritional Sciences, Cornell University, Ithaca, NY 14853, USA.

Materials and Methods

Cell culture, Heat shock treatment and RNA isolation

MEF cells and HeLa cells were maintained in Dulbecco's Modified Eagle's Medium (DMEM) with 10% fetal bovine serum (FBS). Cells were cultured at 37°C with 5% CO_2. Mycoplasma testing was performed routinely and cells tested negative were used in further experiments. Heat shock induction of *Hspa1a* was carried out by incubating the MEF cells in a 42°C water bath for 1 h, followed by recovering in an incubator at 37°C for 2 h. Total RNA was isolated using TRIzol (Ambion) according to the manufacturer's instructions. The RNA concentration was determined on a NanoDrop (Thermo Scientific).

Generation of HeLa cells with knockdown of *METTL5*

The shRNA targeting sequences were cloned into pRSI9-U6-(sh)-UbiC-TagRFP-2A-Puro (Cellecta, CA). Lentiviral particles were packaged using Lenti-X 293T cells. Virus-containing supernatants were collected at 48 h after transfection and filtered to eliminate cell debris. HeLa cells were infected by the lentivirus for 48 h and stable HeLa knockdown lines were generated using puromycin selection (0.5 μg/ml). shRNA targeting sequences are listed below.

METTL5 (target sequence 1): 5′-GCCAGCATCATACAAGTTTCAC -3′;

METTL5 (target sequence 2): 5′-GTCATTCGATACAGTAATTAT -3′;

Scramble target sequence: 5′-AACAGTCGCGTTTGCGACTGG-3′.

RNA oligonucleotides and primers used in SMART assay

HPLC purified RNA oligonucleotides were purchased from Thermo Scientific. The synthetic RNA sequence is derived from the *Hspa1a* 5'UTR. DNA primers ordered from Integrated DNA Technologies were purified by polyacrylamide gel electrophoresis.

RNA oligonucleotides used in this study are as follows:

m^6A positive RNA template: 5'-CGAUCCUCGGCCAGG(m^6A)CCAGCCUUCCCCAG-3'

m^6A negative RNA template: 5'-CGAUCCUCGGCCAGGACCAGCCUUCCCCAG-3'

Primers used for SMART are listed as follows:

A103 of *Hspa1a* mRNA: 5'-AGGGATGCTCTGGGGAAGGCTGG-3'

A12 of *Hspa1a* mRNA: 5'-CCAGATTTGGTTCTGAGTAGCTG-3'

A50 (control site) of *Hspa1a* mRNA: 5'- CGCCGCTCGCTCTGCTTCTCTTGTCTTCGCT-3'

A1640 of 18S ribosomal RNA: 5'-GCAGGGACTTAATCAACGCAAGCTTATGACCCG CACTTACTGGGAAT-3'

A1832 of 18S ribosomal RNA: 5'-AATGATCCTTCCGCAGGTTCACCTACGGAAACCTTG-3'

Crosslinking of synthesized RNA or cellular RNA

250 ng synthesized RNA oligos or 10 μg cellular RNA were diluted in 450 μL immunoprecipitation buffer (50 mM Tris, pH 7.4, 100 mM NaCl, 0.05% NP-40) and incubated with 50 ng or 2.5 μg m^6A antibody (Synaptic Systems) at 4 °C for 2 h. The solution was then cross-linked twice with 0.15 J/cm^2 UV light at 254 nm in a Stratalinker (Agilent). RNA was directly precipitated by adding 1/10 volume of 3M sodium acetate, pH 5.2 and 2.5 volume of cold ethanol and further used in the SAMRT assay.

SuperScript III-mediated SMART assay

The annealing mixture was prepared in a total volume of 6 μL with 50 pmole primer and 250 ng synthesized RNA or 10 μg cellular RNA. The mixture was heated at 90 °C for 1min and incubated at a temperature gradient to cool down slowly (80°C for 1min, 70°C for 1min, 60°C for 1 min, 50°C for 1 min, and then 40°C for 6 min). Then the annealing solution was combined with 2 μL 5×SuperScript III

buffer (Thermo Fisher Scientific), 1 μL 100 mM DTT and 0.5μL SuperScript III enzyme (200 U/μL). The mixture was incubated at 55 °C for 10 min. After adding 0.5 μL 20 μM Fluorescein-12-dUTP (final dUTP concentration: 1 μM), the reactions were incubated for another 5 min at 55°C. Primer extension products were mixed with 10 μL of 2×TBE-Urea buffer and heated for 3 min at 70 °C. Half of the reaction products were resolved on 15% Novex TBE-Urea gels (Thermo Fisher Scientific). Fluorescence signal generated by the extension products was detected by Typhoon 9400 or Typhoon Trio variable mode imager and analyzed by Quantity One 4.6.2. The gels were stained by SYBR-gold dye following fluorescence scanning to visualized the input RNA and probes.

SAMRT assay using distinct RT Enzymes

The reverse transcription assay was prepared with m^6A-crosslinked RNA and 50 pmole primer. The mixture was heated at 90°C for 1 min and cooled slowly as described above. For reaction catalyzed by Tth polymerase, the annealing solution were combined with 1×Tth buffer, 0.5 U/μL of Tth enzyme, 1 mM $MnCl_2$ and heated at 70°C for 3 min. After adding the Fluorescein-12-dUTP (final concentration: 1 μM), the reaction were carried out at 70 °C for 15 minutes. The reaction temperatures for Fluorescein-12-dUTP incorporation and final concentrations for other enzymes are as follows: Superscript II RT (Invitrogen) 20 U/μL, 50°C and AMV RT (Invitrogen) 1.5 U/μL, 50°C. The reaction products were separated using 15% TBE-Urea gels and visualized *via* Typhoon 9400 or Typhoon Trio imager.

Determining the quantitative feasibility of SMART assay

Synthesized RNA oligos with varies m^6A:A ratios from 0 to 100% in the presence of 1 μg total RNA from HeLa cells were used in the assay. The RNA mixture and 250 ng m^6A antibody were incubated at

4°C for 2 h before cross-linked twice with 0.15 J/cm^2 UV light at 254 nm. RNA was precipitated by ethanol and further used in SMART assay. After quantification, the incorporation ratio at each concentration was calculated after normalization to the RT signal at 100% A. GraphPad Prism was employed to plot the points and calculate the standard deviations as well as regression line.

SMART-based detection of m^{6}A in mRNA and ribosomal RNA

For detection of m^{6}A in *Hspa1a* mRNA or 18S ribosomal RNA, 10 μg RNA isolated from MEF or HeLa cells was crosslinked with anti-m^{6}A antibody as described above. Annealing reaction was prepared in a total volume of 6 μL with 50 pmole primer and the m^{6}A-crosslinked RNA. The mixture was heated at 90°C for 1 min and slowly cooled down. Annealing solution was incubated with 2 μL 5×SuperScript III buffer (Thermo Fisher Scientific), 1 μL 100 mM DTT and 0.5 μL SuperScriptIII reverse transcriptase (200 U/μL) at 55°C for 10 min. After adding the 0.5 μL 20 μM Fluorescein-12-dUTP (final concentration: 1 μM), the reaction was incubated at 55 °C for another 5 minutes. The reaction was terminated by adding an equal volume of 2×TBE-Urea buffer and heated for 3 min at 70 °C. Reaction products were resolved on a 15% denaturing polyacrylamide gel, which was imaged and quantified as described above.

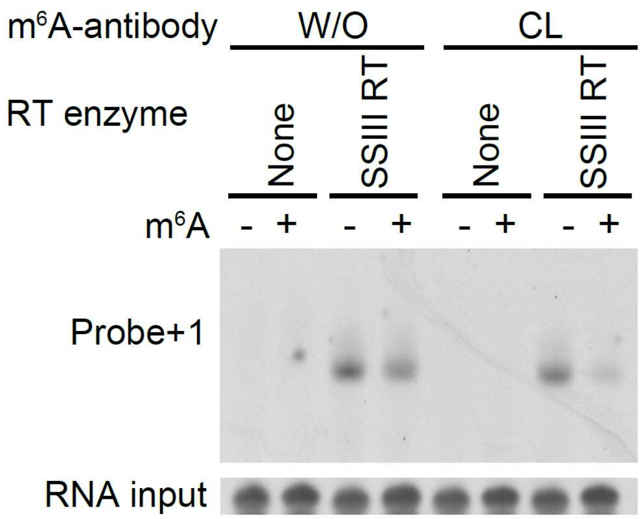

Figure S1. Comparison of SuperScript III-mediated reverse transcription with and without antibody incubation coupled with crosslinking. Synthesized RNA with A or m^{6}A at specific site were used in SuperScript III-directed RT (*left*) or SMART assay (*right*). Related to Figure 1C.

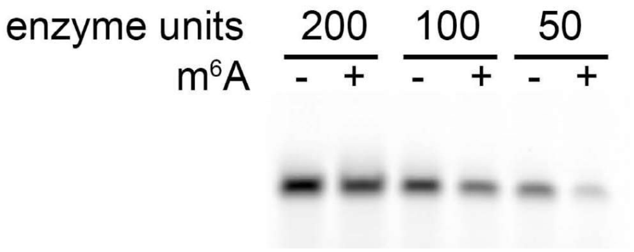

Figure S2. SMART assay was performed using various doses of SuperScript III. Related to Figure 1E.

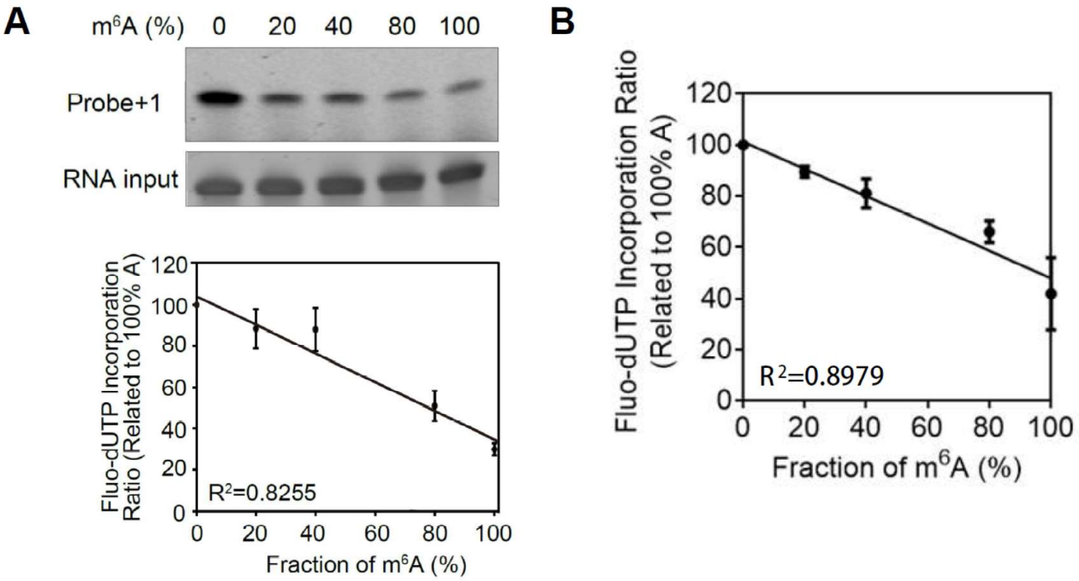

Figure S3. Synthesized RNA with varied ratios of m^{6}A:A incubated with or without cellular RNA were used in SMART assay. (**A**) SMART-based detection of m^{6}A with indicated percentage in synthesized RNA. Primer extension bands were visualized on a TBE gel (upper panel). The 'probe+1' band signals were quantified by normalizing to the signal from SYBR-gold-stained synthesized RNA input in the quantification plot (lower panel). Error bars, mean ± s.e.m. (n=3). (**B**) Quantification plot for SMART-based detection of m^{6}A with indicated percentage in synthesized RNA mixed with HeLa total RNA, Error bars, mean ± s.e.m. (n=2), related to Figure 1F.
